# A Collaborative Data Sharing Platform to Accelerate Translation of Biomedical Innovations

**DOI:** 10.3390/bioengineering12090938

**Published:** 2025-08-30

**Authors:** Zohreh Izadifar, Greg Storm, Amol M. Joshi, Anna Hochberg, Michael Hadjisavas, Gary Rodrigue, Steven R. Bauer, James B. Schmidt, Sita Somara, Anthony Atala, Izabele Heyward, Salil Desai, Joshua Hunsberger

**Affiliations:** 1 Boston Children’s Hospital, Harvard Medical School, Boston, MA 02115, USA; 2 Tripleblind, 800 W 47th St #600, Kansas City, MO 64112, USA; 3 School of Business, Wake Forest University, Winston-Salem, NC 27109, USA; 4 ReMDO, Winston-Salem, NC 27101, USA; 5 p-Chip Corporation, Chicago, IL 60661, USA; 6 Allbright Consulting, Telluride, CO 81435, USA; 7 Wake Forest Institute for Regenerative Medicine (WFIRM), Winston-Salem, NC 27101, USA; 8 Durendal AI Inc., Winston-Salem, NC 27101, USA; 9 College of Engineering, North Carolina Agricultural and Technical State University, Greensboro, NC 27411, USA

**Keywords:** artificial intelligence, biomedical innovations, chain of identity, federated learning, intellectual property, knowledge hub

## Abstract

This perspective article presents an innovative concept for a biomanufacturing Knowledge Hub (KH), designed as a data-driven learning platform supporting the entire lifecycle of biomedical products. By integrating advanced data sharing and processing technologies, the KH aspires to connect patients, bioengineers, clinicians, regulators, companies, and investors to accelerate product development, reduce redundancies, and ultimately fast-track the delivery of biomedical innovations to patients. We discuss current challenges in accessing and sharing data within biomanufacturing and outline novel approaches for building an ecosystem that links data stores, integrates digital twins, and leverages advanced analytics. The KH offers transformative capabilities, enabling the development of new products at a substantial increased speed. It is built as a secure, quantum-resistant platform that encrypts data and allows access through advanced algorithms, creating an intelligent, collaborative environment. Users can harness collective knowledge to enhance products, launch innovations, integrate technologies, and unlock revenue opportunities based on data quality and usage. This KH aims to revolutionize biomanufacturing, offering unprecedented opportunities for innovation, better patient outcomes, and commercialization with far reaching applications beyond biomanufacturing in the future.

## 1. Introduction

Despite great progress in biomedical engineering over the last 30 years, the number of products that have successfully been approved or commercialized for clinical practice remains significantly limited. This is because of the complexity of the technologies and processes involved in developing, manufacturing, validating, approving, and commercializing biomedical innovations, which makes the bench-to-bedside path challenging and long [1]. Additionally, the need to preserve the intellectual property (IP) benefits and commercial advantages adds another level of complexity to biomedical products life cycle. Currently, academic research articles are the primary outlets for sharing data on scientific developments and, occasionally, the results of clinical trials of biomedical products. The U.S. government’s ClinicalTrials.gov website is currently the primary public repository of clinical trial studies from around the world. However, this database lacks critical translationally relevant information on trial outcomes, regardless of success or failure. In many cases, results are shared only briefly—if not at all—and little to no detail is provided on why certain trials are terminated, fail, or are abandoned. This lack of transparency makes it impossible to fully learn from past clinical trials, leaving the field prone to repeating costly studies over and over again. Information and data on important phases and processes of the product translation, including biomanufacturing, scale up, preclinical derisking, and regulatory and commercialization processes, remain largely unpublished, nontrackable, undocumented, or stored in isolated databases with limited access [2]. Curated repositories with indexable and verifiable sources are severely lacking. Regardless of the success or failure of the products, translational and commercialization knowledge, as well as information generated during the translation process, are of high value to researchers and industry stakeholders to identify pitfalls, pain points, and challenges. It is crucial to strategically centralize and leverage decades worth of generated knowledge and information to accelerate successful translation and commercialization of biomedical products, especially for Tissue Engineering and Regenerative Medicine (TERM) innovations. The current state of minimally shared—if not at all— knowledge and data has resulted in duplicated effort, decades of slow progress, repetitive failed trials, or parallel struggles with the same technical, regulatory, or commercialization challenges. These hurdles cost the public and private sectors millions of dollars in addition to the continuous economic burden of persistent medical conditions biomedical products are aiming to resolve [3].

For example, TERM products that showed early promise, such as bioengineered bladder, trachea, skin, cartilage, and certain stem cell–based therapies, still face challenges in advancing to clinical applications, even after more than two decades. Common reported setbacks, such as mixed long-term clinical trial outcomes, variability in patient responses, scale-up manufacturing difficulties, regulatory uncertainties, and cost-effectiveness and reimbursement issues, still pose major hurdles in the translation of TERM products. Detailed knowledge about these challenges and failures has often remained siloed or confidential for various reasons, which has hindered progress in the field. Sharing this valuable information could have served as a feedback loop to the research and development efforts in academia and industry, enabling early adjustments that increase the likelihood of success in downstream translational and commercialization barriers. As such, it is reasonable to recognize the lack of a constructive data-sharing platform as a major obstacle in the field, slowing down breakthroughs in the translation and commercialization of biomedical products. This perspective article seeks to lay out the conceptual framework for a data sharing platform, which we term Knowledge Hub (KH), that we envision will accelerate the translation of biomedical innovations.

## 2. Data Sharing Challenges in the Translation of Biomedical Products

The development of technologies to manage sensitive information has emerged as one of the most significant advancements in the field of data analytics in recent years. Advancement in biomedical engineering has a growing need to combine medical data from multiple sources to generate valuable insights [4]. Concerns with data protection are increasing. According to the United Nations Trade and Development (UNCTAD), in 2021, 71% of the countries worldwide had legislation to guarantee data protection and privacy, while 9% had draft legislation [5]. Table 1 presents a few examples of regions with heavy data privacy regulations.

Commercializing new biomedical products involves considerable challenges due to the bureaucracy and ethical considerations associated with the necessary regulations of data-sharing [10]. Traditional data-sharing models face many limitations due to security concerns, prompting the exploration of alternative solutions. For example, centralized data storage systems pose a significant risk since databases are controlled by a single entity, making them vulnerable to single points of failure, data breaches, and unauthorized access [11]. Security is another major concern; traditional models typically rely on perimeter-based defenses, leaving them susceptible to cyber attacks [12]. New models must ensure the security and integrity of sensitive data in transit and at rest across multiple systems and platforms. The lack of transparency complicates the data-sharing process, making it difficult to trace the origin and flow of data; thus, new platforms should enhance transparency to address discrepancies, fraud, and mistrust [13]. Additionally, data silos hinder collaboration and interoperability, as organizations often isolate data. New platforms need to break down these silos to enable seamless connectivity and foster transformative collaborations and innovations [14]. Inefficient intermediaries further complicate data sharing by introducing delays and potential failure points; new models should minimize reliance on these intermediaries to streamline the process [15]. Concerns about data privacy and the reluctance to share sensitive information due to IP benefits also complicate collaboration. Issues surrounding ownership and control over shared data must be addressed, ensuring individuals retain ownership of their data and privacy while encouraging secure data exchange, collaboration, and collective knowledge extraction.

Here are three concrete examples of cross-border data-sharing challenges across three different sectors including automotive, public health/tech, and disaster risk reduction to illustrate the need for improved data sharing platforms that can address these real-world examples. In the automotive sector, Germany and China faced significant obstacles in transferring vehicle-generated data due to Chinese regulatory restrictions and political sensitivities, leading both countries to sign a 2024 Memorandum of Understanding to facilitate limited carmaker data exchanges while navigating security concerns. In public health and technology, the COVID-19 pandemic exposed how national contact tracing apps were often siloed, with limited interoperability across borders; while Ireland and Northern Ireland achieved a rare cross-border solution, broader EU-wide integration faltered due to divergent legal frameworks and technological standards. In disaster risk reduction, recurring natural disasters along the Italy–France border highlighted the difficulty of sharing critical risk data, with efforts hindered by lack of mutual trust, fragmented strategies, and uneven technical capacities—barriers that persist despite local collaborative efforts. These examples underscore the urgent need for robust, trusted platforms that enable secure and effective cross-border data sharing.

Finally, the complexity and variability of compliance requirements present obstacles to cross-border data sharing due to differing regulatory frameworks [16]. New models must incorporate standards to harmonize data types and compliance requirements. Ensuring the quality and integrity of shared data is crucial; therefore, new platforms should implement mechanisms to verify and assess data quality throughout their lifecycle, relying on established standards for designing experiments and recording data.

## 3. Data Sharing Technology Solutions for Biomedical Innovations

Recent advancements in data architecture—such as data fabrics, data meshes, and zero-trust data sharing frameworks—have significantly improved the management and interoperability of biomedical data across institutional and geographic silos. In parallel, privacy-preserving technologies such as federated learning, trusted execution environments (TEEs), and fully homomorphic encryption (FHE) are enabling data and model sharing without compromising security or confidentiality. Among these, blockchain technology (BT) has emerged not only as a secure and tamper-evident digital ledger system but also as a transformative platform for building decentralized, autonomous data-sharing ecosystems that enable new models for knowledge co-creation, IP governance, and digital commerce.

### 3.1. Blockchain as Foundational Infrastucture for the Knowledge Hub (KH)

The modern concept of blockchain was popularized by Bitcoin in 2008 [17], but the foundations trace back decades earlier to innovations in cryptographic hashing, Merkle trees, Byzantine fault tolerance, and digital timestamping services, such as the early “proto-blockchain” evident in The New York Times classified ad cryptographic logs [18] (Haber & Stornetta, 1991). Blockchain is a specific type of distributed ledger technology (DLT) in which data is stored in blocks, cryptographically linked in chronological order to form an immutable chain shared across a decentralized network. Each node maintains a synchronized copy of the ledger, secured through consensus mechanisms such as Proof of Work, Proof of Stake, or newer Proof of Authority protocols.

Contrary to simplistic narratives, blockchain is not synonymous with cryptocurrency, nor is it exclusively a digital ledger. With the advent of programmable blockchains such as Ethereum, blockchain evolved into a decentralized computing substrate for web applications—now foundational to the development of Web 3.0 [19,20]. Web 3.0 applications leverage smart contracts—autonomous scripts that self-execute when pre-specified conditions are met—to enable decentralized finance (DeFi), identity management, digital governance, and data sharing systems without central intermediaries.

Blockchains come in three architectural forms: permissionless (open to anyone, e.g., Ethereum), permissioned (access restricted, e.g., Hyperledger), and hybrid models that combine public transparency with private control—particularly useful in regulated industries like biomanufacturing. In the context of the KH, a hybrid blockchain could facilitate both public auditability and fine-grained access control for sensitive clinical, genomic, or process data.

Smart contracts in this framework could govern: (1) Data usage rights, automatically enforcing licensing conditions and payments; (2) Model deployment, where a contract validates input/output constraints and triggers model execution only under compliance conditions; (3) Revenue sharing, where proceeds from downstream applications (e.g., diagnostics or therapies built using KH-derived data or models) are automatically distributed to data/model contributors.

This programmable trust infrastructure directly supports the tokenization of digital assets—ranging from raw data to trained AI models—and the creation of data-backed tokens or model derivatives that can be exchanged, licensed, or collateralized. IP can be embedded into blockchain-based registries using cryptographic proofs of authorship, licensing metadata, and traceable transaction histories [21,22].

By integrating BT into the KH, participating entities—researchers, manufacturers, regulators, and patients—can maintain sovereignty over their contributions while collaborating in a shared innovation space. Each data transaction can be recorded, timestamped, cryptographically secured, and tied to smart contracts that govern consent, commercial use, and attribution. This trustless yet verifiable environment can alleviate long-standing tensions between open science and proprietary protection, particularly among for-profit actors historically reluctant to share translational data.

**Figure 1:**
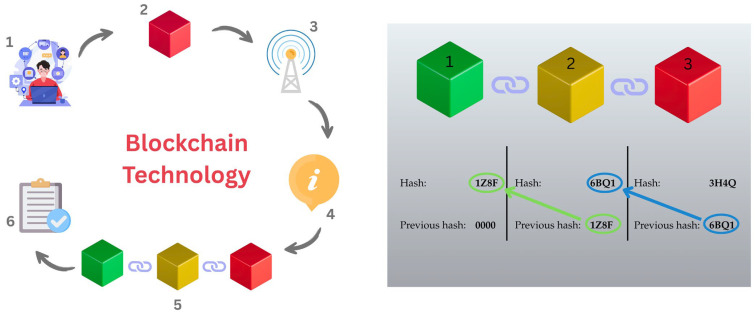
Blockchain workflow and structural integrity for trusted data exchange. The (**left**) panel depicts the end-to-end process of blockchain transaction flow in six steps: (1) data generation by users or devices, (2) encapsulation into a block, (3) broadcasting to the network, (4) validation by consensus mechanisms, (5) addition to the distributed ledger, and (6) confirmation and archival. The (**right**) panel shows how each block contains its own cryptographic hash and the hash of the previous block, forming an immutable chain (1,2,3). This dual-layered model—process and structure—underpins blockchain’s potential for secure, transparent, and verifiable data exchange in biomanufacturing and within the broader KH ecosystem. The first block in the chain, known as the genesis block, is uniquely identified by having no predecessor and is typically anchored with a null or zero-valued hash.

In effect, blockchain transforms the KH from a static repository into a dynamic digital marketplace for biomedical knowledge assets. It provides the foundational scaffolding for a new generation of secure, decentralized biomedical innovation ecosystems, accelerating translational research while aligning incentives across the value chain. Figure 1 presents a blockchain workflow and structural integrity for trusted data exchange.

The Inter-Blockchain Communication Protocol (IBC) promotes interoperability and collaboration between different blockchain ecosystems or silos of data.

- Connection Establishment: Two blockchains establish a connection, which involves agreeing on a common set of rules for communication. This set of rules is established when the blockchain is initially set up and helps to provide a framework for a high level of security and integrity.
- Channel Creation: Within the established connection a channel is created for sending the data. Note: Specific channels can be created for different types of messages or transactions.
- Message Exchange: Blockchains can send packets of data (messages) to each other over the established channels. Each message can carry information about transactions, state changes, or other relevant data.
- Verification: Messages are verified using cryptographic proofs to ensure that they are legitimate and have been sent from an authenticated source. These authenticated sources are established by the developers and the stakeholders.
- State Updates: The receiving blockchain is updated based on the information contained in the incoming message.

### 3.2. Federated Learning

Federated Learning (FL) is a machine learning approach that emerged in healthcare as a solution for privacy preservation, allowing a shared model to be collaboratively trained across decentralized devices or servers that hold local data samples without exchanging them. Instead of sending raw data to a central server for model training, the model is sent to the local data sources, and the training is performed on the device or server itself. The updates to the model are then aggregated, typically at a central server to improve the global model. This process is carried out iteratively, and the global model becomes increasingly accurate without exposing raw data [23].

**Figure 2:**
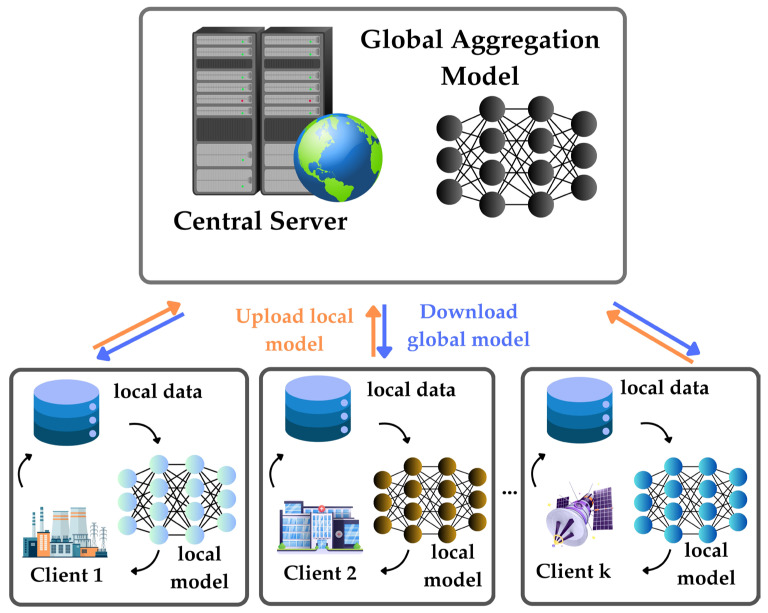
A schematic of a FL framework: in each k iteration, a client downloads the latest global model from the server, trains it with their local datasets, and then proceeds to upload the updated local model back to the server. The servers implement model aggregation on the new local model, creating an updated global model.

Figure 2 shows a schematic of an FL framework. In each k iteration, a client downloads the latest global model from the server, trains it with their local datasets, and then proceeds to upload the updated local model back to the server. The servers implement model aggregation on the new local model, creating an updated global model. This learning meets the critical need for data privacy while efficiently managing many remote devices [23,24].

The privacy advantages of maintaining the data locally are substantial. Data that does not leave the organization’s firewall can be kept secure and private per the organization’s policies and tools. Local operations can manage opt-in/opt-out decisions, and entire organizations can decide whether to allow their data to be consumed during a specific operation. In advanced models, “federated” data allows for distinct computational advantages (where some of the computing can be distributed to the participating data nodes). Operationally, processes can also be streamlined—data collaboration agreements can be automated, liability can be limited (no one has all the data), and specific uses can be governed with tools rather than contracts. Currently, there are several operational “federated data” exchanges, including Kidsights^TM^ [25] and Mayo Clinic Platform [26].

Some key characteristics and components of FL that can benefit the data-sharing KH for biomedical engineering are:

- Decentralized Model Training: FL enables model training to occur on local devices or servers where the data resides, rather than centralizing all data on a single server.
- Model Distribution: The initial model or its parameters are distributed to participating devices. Each device trains the model locally using its data and then sends only the model updates (gradients) back to the central server.
- Aggregation: The central server aggregates the received updates from multiple devices to update the global model. Common aggregation methods include simple averaging or more sophisticated methods like weighted aggregation.
- Privacy-Preserving: Raw data never leaves the local device, and only the model updates are shared, helping address privacy concerns associated with centralized models.
- Iterative Process: The process of distributing the model, training locally, and aggregating updates is performed iteratively. The global model improves over time as each local model contributes information.
- Communication Efficiency: FL reduces the need for large-scale data transmission since only model updates are exchanged. This can be beneficial in scenarios where bandwidth or communication costs are significant concerns.
- Personalization: FL allows for personalized model updates based on local data. This is especially useful in applications where individual user preferences or characteristics are important.
- Edge Computing Integration: FL is well-suited for edge computing environments where devices at the edge of the network (e.g., smartphones, IoT devices) can participate in model training without relying heavily on centralized cloud servers.
- Robustness: The distributed nature of FL can enhance model robustness. Local models may adapt to diverse data distributions, leading to a more robust global model.

FL is particularly relevant in applications where data privacy is crucial, such as healthcare, finance, and mobile applications. Due to the highly sensitive nature of medical data, any breach can have severe consequences for patients and industry partners, causing inefficient treatment and generating economic setbacks to institutions [27]. Therefore, improving healthcare data confidentiality is essential. Federated systems allow for collaborative model training without compromising individual data privacy and security while enforcing global data privacy rules.

Even though FL is an effective framework for data privacy, the possibility of data leakage still exists. For this reason, privacy-enhancing computing techniques can be incorporated into the learning process, including homomorphic encryption and multi-party computation (MPC). These techniques have been used successfully in healthcare for medical data. Truhn et al. [28] applied somewhat homomorphically encrypted FL across a variety of clinically relevant tasks in cancer image analysis, utilizing multicentric datasets from radiology and histopathology. The researchers demonstrated the capability to securely train the FL models on multi-institutional data while preserving patient privacy. Sun et al. [29] created a ML model to predict the risk of Diabetes Mellitus by applying a secure MPC to avoid the risk of leaking the original feature for the FL regression algorithm during training.

One example of a privacy-preserving framework to improve learning is the TripleBlind Technology, which offers a secure, private collaboration system that operationalizes and improves Federated Learning. TripleBlind is delivered through software that is installed at each of the federated nodes. The software then empowers each node to perform a range of collaborative tasks, on all or some subsets of the data available at the collective nodes (i.e., all or some designated subset of the data). This system has been validated by HIPAA Expert Determination to perform all operations in a “de-identified” manner; thus, preserving appropriate privacy for everyone. The Application Programming Interface (API) is set up to automatically de-identify the data in line with the HIPAA Expert Determination method rather than using the traditional approach of relying on an expert to de-identify each dataset one by one. This approach of automatically assuring privacy delivers several significant benefits including the speed with which the collaboration can happen (i.e., data can be available for computation in real-time) and the breadth of usage that can be explored (i.e., users can employ the data regardless of institution, organizational affiliation, etc.). Once privacy is assured, data becomes much more “liquid” across organizational boundaries. The TripleBlind system supports several key functions, such as:

- Data Discovery. There is a primary graphical user interface (GUI) that allows data users to discover data schematic information and perform Exploratory Data Analysis (EDA) tasks.
- Data Management. Each data owner has complete Digital Rights Management (DRM) capability for their data. Each owner can determine who uses their data and for what purpose.
- Data Usage Management and Audit. TripleBlind ensures that data is only used for authorized purposes (meaning no unauthorized secondary usage of data is allowed).
- Data User Functionality. A wide range of functionalities are available such as:
  - Logical data aggregation across data nodes, both horizontally (same data at multiple locations) and vertically (same subject at multiple locations). In the case of vertically arranged data, the system supports sophisticated private record linkage functionality.
  - Data harmonization tools. Disparate data can be “pre-processed” into the appropriate format for a given analytical purpose.
  - Analytical tools. The system supports everything from simple queries to sophisticated dashboards.
  - Machine Learning/Artificial Intelligence (ML/AI) tools. The system supports multiple python data science libraries such as SciKit Learn, PyTorch and Pandas.
  - Model delivery. ML/AI models frequently need to be delivered to data in organizations other than the organization in which they were trained, on data that is private to the owning organization (i.e., a diagnostic at a hospital). TripleBlind ensures the appropriate privacy for both the data owner and the model owner.
  - Audit Trail. TripleBlind is a secure, closed system (versus an open-source project) that insists that all assets (datasets, models, and algorithms) have cryptographically enforced identifiers. All users have access to credentials. These immutable identifiers are used to record a complete audit trail for every interaction on the system.
- Multi-modal data capability. The system operationalizes any type of data that can be stored electronically—tabular, text, image (i.e., dicom, etc.), video, etc.
- Data Location flexibility. TripleBlind software can run on any server capable of running Linux. Therefore, any data location is accessible—in the cloud, across cloud providers, on-premise, or in space—as long as there is an internet connection available.
- True Data Mesh. The TripleBlind router makes any data useful to any user in the collaboration, effortlessly. Users use the dataset names available via the data discovery tools; the TripleBlind router takes care of the server-to-server (peer-to-peer) connectivity necessary to accomplish the required computation.

The TripleBlind toolset also offers practical benefits. The data can be available to researchers in nearly real-time. Because privacy is automatic, the data can be used simultaneously as it is being created, in addition to automatically enforced data de-identification. These automated processes will direct the expenses that are normally associated with data de-identification elsewhere, alleviating the costs of data curation on a large scale [30].

### 3.3. Homomorphic Encryption and Other Approaches to Data and Data Model Security

Fully Homomorphic Encryption (FHE) allows computations on encrypted data without decryption, producing encrypted results that match operations performed on unencrypted data once decrypted. FHE supports a wide range of algorithmic operations on ciphertexts, making it useful in high-security areas like cloud computing and medical data processing, as sensitive data remains confidential during operations. FHE’s versatility extends to secure data analysis and private information retrieval, enabling data processing without exposing underlying information. Concrete machine learning (ML) is a Privacy-Preserving Machine Learning open-source set of tools built on top of Concrete, an open-source FHE compiler. It simplifies the use of fully homomorphic encryption (FHE) for data scientists so that they can automatically turn machine learning models into their homomorphic equivalents and use them without knowledge of cryptography. FHE faces challenges: its operations are slower than traditional encryption, requiring specialized knowledge for implementation. Despite these hurdles, FHE is a groundbreaking approach to data security, supporting computations on encrypted data while preserving privacy, though practical application continues to evolve through active research and development. It should be noted that Concrete ML supports Tree-Based Models (Decision Trees, Random Forest, XGBoost), Linear Models (Linear Regression, Logistic Regression, Generalized Linear Models, Suport Vector Machine, ElasticNet, Lasso, Ridge), and Neural Networks (Multi-Layer Perceptron, Custom Neural Nets via Tourch, Tensor Flow, Open Neural Network Exchange).

FHE has been successfully used to encrypt health data. Sun et al. used FHE for data security in a mobile healthcare network, which consisted of the following sections: wearable device, preprocessing, cloud server, and physician diagnosis. The model allowed secure computation of average heart rate, heart rhythm disorders, and chi-square tests [31]. Malik et al. employed FHE within a Deep Learning model to diagnose diseases. The system was able to predict with 95% accuracy between 90 diseases based on a survey of possible symptoms [32]. Shaikh et al. used FHE to safely analyze Electrocardiogram (ECG) data [33].

Other security and privacy approaches that can be used to support the KH data and models security and privacy include secure multi-party computation (MPC), differential privacy, federated learning and others to name a few. MPC, such as Secure Multiparty Quantum Computation for post-quantum resilience and Confidential Computing, which employs CPU hardware enclaves, or trusted execution environments, that can protect the security and privacy of both data and algorithms. In a distributed network, secure MPC involves computing a probabilistic function on a global input in which different clients hold onto their localized input node. The objective is to maintain the independence of inputs, ensure the correctness of output, and guarantee that only needed information is shared with a client in the computations [34,35]. Differential privacy induces noise with datasets to evaluate trend analysis without revealing the details of the data [36]. Federated learning, as discussed earlier, trains AI models at a high-level on localized datasets obviating the need to pool data to a central location, thereby retaining privacy of the employed data nodes [37]. These methods can be selected based on the data types, data heterogeneity, volume and user privacy requirements. In addition, a combination of these technologies can be implemented considering computational overhead for timely decision-making as well as their integration with legacy healthcare systems.

### 3.4. New Strategies for Intellectual Property

A major inhibitor for research and development (R&D) collaboration and commercialization in biomanufacturing is the fragmentation of intellectual property rights (IPRs) across the broad range of prospective market participants [38]. For example, a large and ever-widening array of biopharma companies, biotech startups, university R&D labs, and individual inventors hold the critical patents for key processes and core products required to manufacture biomaterials at scale and at exacting levels of quality and purity. Typically, no single entity owns all of the necessary IPRs associated with these processes and products [39]. As a result, it is burdensome to identify and source all of the complementary IPRs needed to commercially deliver new processes and products within a reasonable time horizon [40]. Hence, the substantial costs of first finding others’ existing IPRs and then avoiding the risks of infringing on these IPRs frequently prevent biomanufacturing market participants from achieving their commercial aims [41].

One effective solution for directly addressing the central issue of fragmented IPRs is the formation of patent pools. A patent pool is a “contractual relationship that aggregates IPRs among multiple entities to develop and commercialize new technology-based products. This contractual relationship requires explicit approval from regulatory authorities over anticompetitive concerns.” [42]. Current regulations and legal precedents in the US and around the world generally view patent pools as procompetitive as long as upstream licensors do not unduly restrict downstream licensees [43]. In other words, any interested party should be able to license the pooled patents in a reasonable and non-discriminatory manner with clear fees, terms, and conditions [42]. Noteworthy examples of successful patent pools include consumer electronics (MPEG-2 and DVD), mobile telecommunications (3G and 4G), and the Medicines Patent Pool (MPP) [41,42,44].

The main benefits of patent pools in biomanufacturing include reduced transactions costs and accelerated innovation; the introduction of government-approved patent pools streamlines access to essential technologies and reduces the threats of unproductive litigation [44,45,46]. For example, negotiating individual patent licenses can be costly and time-consuming. Patent pools simplify this process by offering bundled access or one-stop shopping, saving resources for both patent holders (licensors) and licensees [45]. Patent pools speed up innovation by encouraging direct knowledge sharing among researchers. Instead of expending effort on navigating patent restrictions, researchers can more easily and rapidly build on proven solutions licensed out by others through the established pool.

Although the concept of patent pools is relatively new in biomanufacturing, their potential is significant. This is primarily because patent pools offer a proven way to overcome the problem of fragmented IP rights that are likely to impede knowledge sharing. Industry-led initiatives such as the MPP, which focuses on pharmaceutical patents, provide a model for how similar strategies could be applied to biomanufacturing contexts [44]. By aggregating essential patents, offering standardized licensing terms, and fostering cooperation among stakeholders, patent pools can accelerate the development of biopharmaceuticals. Prior studies show that industry associations and technical standards bodies are an important precursor to the eventual formation of a patent pool [47,48,49]. Historically, such associations and standards were less oriented towards concerted action to form patent pools. However, by bringing together market participants and defining the key technological elements, emerging industry-led initiatives such as the Regenerative Medicine Manufacturing Society (RMMS) can play a vital role by driving the subsequent formation of patent pools and promoting a more robust innovation ecosystem in biomanufacturing.

There are additional legal and regulatory risks for a biomanufacturing KH patent pool which could include (1) antitrust scrutiny across jurisdictions, (2) disagreements over essentiality and standardization, (3) territorial fragmentation of IP, (4) compulsory licensing regimes, (5) overlap with trade secrets/data privacy, and (6) export control restrictions. We envision RMMS serving as a neutral third party to assist with mitigation of these legal and regulatory risks. For instance, in the area of Antitrust & Competition Law, where the risk is being viewed as an anti-competitive cartel if licensing terms restrict market access or innovation, RMMS can act as a neutral clearinghouse ensuring all licensing is on FRAND (Fair, Reasonable, and Non-Discriminatory) terms, provide transparent governance (e.g., published participation rules, open eligibility), and establish an independent antitrust advisory panel with representatives from U.S., EU, and Asia-Pacific legal experts to pre-clear practices. In the area of Patent Essentiality & Standard-Setting Risks, where ambiguity exists in defining which patents are “essential” to biomanufacturing standards (e.g., iPSC workflows, bioreactors), RMMS can create a Technical Standards Committee, commission independent essentiality reviews, and enable tiered pools to prevent over-inclusion. In the area of Cross-Border Licensing & IP Fragmentation, where territorial patent rights complicate enforcement, RMMS can develop a multi-jurisdictional licensing framework with regional sub-pools (U.S., EU, Asia), partner with WIPO to align licensing templates internationally, and maintain a global patent landscape database within the Knowledge Hub. In the area of Compulsory Licensing & Public Health Exceptions, where governments may override licenses for critical therapies, RMMS can assist by embedding flexible licensing clauses (e.g., reduced royalties in emergencies), positioning RMMS as a trusted regulatory partner, and creating a Public Health Sub-Fund to support equitable access.

Further, in the area of Data Privacy & Trade Secret Conflicts, where proprietary process data and algorithms may intersect with GDPR/HIPAA compliance issues, RMMS can separate governance for patent and non-patent data assets, apply privacy-preserving technologies such as homomorphic encryption or secure multi-party computation, and provide standardized Data Sharing Agreements tailored to global contexts. In the area of Export Control & National Security Restrictions, where biomanufacturing technologies may be considered dual-use, RMMS could conduct export control screenings with legal experts, implement segmented regional licensing frameworks, and proactively engage with U.S., EU, and other national security regulators to ensure compliance. In sum, RMMS could serve as a neutral third party to provide the following: (1) Convening—bringing together industry, academia, regulators, and governments in a pre-competitive space; (2) Governance—acting as the trusted steward for transparency, accountability, and neutral decision-making; (3) Standards Development—coordinating essentiality reviews, FRAND licensing, and data sharing protocols; (4) Regulatory Interface—proactively engaging with antitrust authorities and regulators to reduce litigation risk; and (5) Knowledge Hub Integration—embedding patent pools with secure, privacy-preserving digital infrastructure for both IP and data.

## 4. Knowledge Hub Capabilities for Advancing Biomanufacturing

KHs of translational regenerative medicine can be designed as centralized platforms to facilitate the sharing and integration of data across various stakeholders, including researchers, clinicians, and regulatory bodies. These hubs will leverage ML and other forms of AI to organize, analyze, and disseminate large volumes of data, making it more accessible and actionable. KHs represent a transformative approach to data sharing in regenerative medicine, fostering innovation and improving patient outcomes through collaborative research and development.

Data could be collected from a range of sources such as academic, industry, government, hospitals, and not-for-profit organizations that will be integrated into the data-sharing model platform in a secure and encrypted setting where data contributors have control and possession on access to and IP of their datasets. Interested stakeholders could also contribute data from regulatory submissions to various global regulatory authorities while protecting proprietary information. The encrypted data will be employed by data learning algorithms to build an intelligent KH that users/members can learn from existing data and shared knowledge to improve their own products, biomanufacturing and, regulatory approval processes, design and execute efficient trials, accelerate new products launch, integrate new technologies, solve once unsolvable problems, and also offer up new revenue streams for their research and data.

**Figure 3:**
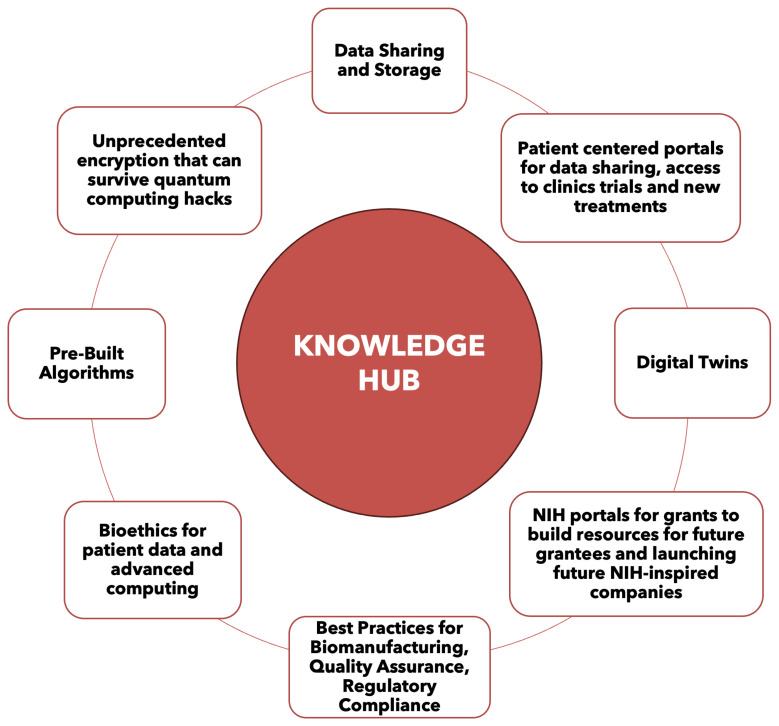
KH capabilities that lay out plans to include data storage, data sharing, access to pre-built algorithms, best practices for biomanufacturing, unprecedented encryption, bioethics for patient data and advanced computing, patient centered portals for data sharing and access to clinical trials and new treatments, and NIH portals for grants to build resources for future grantees to build off of as well as launching future NIH-inspired companies.

Figure 3 outlines the KH’s capabilities, including data persistence, algorithm access, biomanufacturing best practices, advanced encryption, bioethics, patient advocacy portals, clinical trial databases, real-time and cross-institutional data sharing, and National Institutes of Health (NIH) grant portals. These elements aim to build resources for future grantees and NIH-inspired ventures.

Patient Advocacy Portals will enable patients to share data with medical centers conducting clinical trials and access new Food and Drug Administration (FDA)-approved treatments. Inspired by the European Health Data Space model, this system offers secure, encrypted data sharing, empowering patients to engage in finding cures and learning about treatments [50]. Patients can opt into updates on new treatments and standards of care using built-in features in electronic health records systems (e.g., EPIC and CERNER) that connect patients and their data to the KH through a clinical trial advocacy portal. This will allow clinicians and patients to stay informed about active clinical trials and control of access to their health record data. Medical centers could pay membership fees to access this clinical trial advocacy portal, offering the potential for monetization.

Specialty-wide clinical trial databases will be another feature of our KH. This concept has been recently proposed in ophthalmology [51], but we envision expanding this to many different specialties. The advantage we see with this approach is that it will offer unprecedented ways for specialty groups to collaborate and develop new cures, share the best practices, and create standards for datasets.

Real-time knowledge sharing inspired by AbbVie’s bioanalytical unit [52], will be developed, which supports faster insights and troubleshooting for drug studies. Expanding this model to thousands of users, the KH would enable rapid, flexible learning. Additionally, incorporating real-time wearable health data [53] offers opportunities for collective learning without sacrificing data privacy, fostering unprecedented advancements.

Biomanufacturing process development and control will remain a foundational imperative in the implementation, deployment, scaling and delivery of TERM products and related therapeutics. This relevance is driven by the extensive and complex heuristics of these biomanufacturing processes that are inherent to the complexity of biological and genetic systems. Consequently, manufacturers are focused on acquiring and understanding a full command of all potential tunable parameters in these biomanufacturing processes. This is particularly relevant in instances where cell lines and genetic systems exhibit hypervariability in their phenotypes and efficacies influenced by biomanufacturing conditions and treatments. These conditions span parameters across genetic modification, cell manipulation, delivery, administration, storage, passaging, propagation and differentiation, including analytical technologies to name a few. Generally, biomanufacturers recognize that subtle or ostensibly minor variances in a single tunable parameter in their processes can cause significant variability in the quality, consistency and efficacy of the output therapeutic and consequently, patient outcomes. Collectively, establishing a multi-contributor and shareable KH across the ecosystem of stakeholders will address. the pressing need for a comprehensive process control. The intent is to integrate data related to biomanufacturing processes, design inputs and outputs, clinical protocols and patient outcomes to enable the sharing of best practices for enablement of process improvement and scaling across the industry.

**Figure 4:**
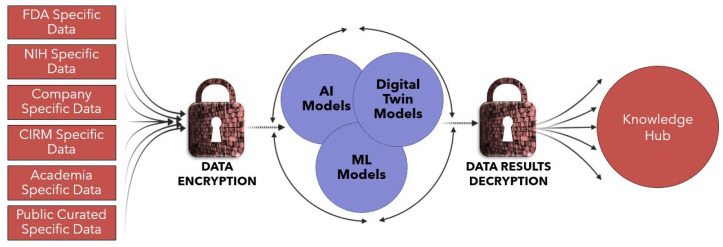
Representation of data encryption for the KH. All private data will be encrypted to train the AI/ML and Digital Twin Models.

Cross-institutional data-sharing platform will use advanced encryption, such as FHE, to protect patient privacy while enabling collaborative data sharing (Figure 4), as demonstrated in AI-based ocular imaging studies [54]. FHE is a revolutionary approach to secure data processing, enabling computations on encrypted data while preserving privacy that is production-ready and will be offered in this KH to ensure encryption that can withstand future threats from quantum computing. This platform addresses issues like those seen during COVID-19, enhancing coordinated public health responses. A future pandemic could be predicted using the KH’s ability to detect unusual patterns—such as a spike in emergency room visits—by aggregating and analyzing real-time data across health systems. Once flagged, the platform could rapidly coordinate a response by activating a curated network of companies, researchers, and public health experts to accelerate diagnostics, treatments, and containment strategies.

KH as a NIH or FDA Portal. The NIH’s Data Sharing Policy [55] and roadmap aim to enhance scientific research’s transparency, reproducibility, and utility by making NIH-funded research data accessible to researchers, policymakers, and the public. The KH could act as an NIH Portal for sharing this data, supporting the launch of NIH-inspired companies to translate research into patient treatments. This aligns with the NIH’s commitment to advancing science and public health through collaborative data sharing. The KH addresses a gap in the NIH roadmap by providing a centralized data-sharing platform to support these goals.

FDA recently released a roadmap for reducing animal testing in preclinical safety studies [56] using New Approach Methodologies (NAMs) data, such as AI-based computational models and in vitro microphysiological systems. Development of a comprehensive data repository including existing animal and human databases, i.e., Integrated Chemical Environment [57] and US Tox21 program [58,59] is one of the key FDA’s implementational steps with the prospect of expansion to other private and/or international databases. KH could substantially facilitate this effort by not only securely and reliably curating databases from national and international databases, but also by providing knowledge extraction capabilities (i.e., digital Twin Models) that will accelerate regulatory evaluation processes, lower R&D costs and bench-to-bedside timeline.

Digital Twin Models of biomanufacturing processes will be developed to address different domains of TERM products [60]. Digital Twin Models are virtual representations or replicates of products, processes, or physical entities created using a combination of physical and analytical data, networks, and computational capabilities to model behaviors, processes, products, and systems. These models can be dynamically visualized, analyzed, monitored, and optimized throughout their lifecycles. In biomanufacturing, digital twins offer tremendous potential for simulating clinical trials and product biofabrication and development processes in silico, using data from similar products or processes to optimize outcomes, reduce costs, and shorten timelines.

For example, a digital twin of the 3D bioprinting process will be developed for specific tissue constructs. This will involve combination of digital data of the tissue architecture along with biomaterials that constitute different bioinks to fabricate specific tissue types such as osteochondral (bone-cartilage) lineages [61]. Anatomical shapes scanned via computer tomography (CT) imaging will be securely exchanged over this KH with unique designations for patient privacy. Further, biomaterials source and quality will be verified using secure BC to eliminate spurious material constituents and ensure highest grade of material usage. Bioprinters will be secured via industrial-internet-of-things (IIoT) sensors to optimize process parameters [62] and material usage such that patient specific tissue constructs will be fabricated for clinical implementation. AI algorithms will be utilized to predict failures in material deposition and process parameters [63]. Digital twins of clinical trials can also be developed to model processes involved in the design and execution, while digital twins of biomolecules, cells, and tissue systems—integrating multi-molecule datasets—can be especially valuable for cell and gene therapy designs and bioprocessing.

In the KH, customers can query digital twins to perform gap analyses, compare products, and optimize clinical trials and biomanufacturing processes. These simulations aim to improve biomanufacturing success rates and advance therapeutic development by reducing redundancies in research, manufacturing, and regulatory processes. As a result, the time spent on drug and therapeutic development and commercialization could be significantly reduced, potentially saving years or even decades. By consolidating data on biomanufactured products and clinical outcomes, KH’s federated data-sharing model, powered by secure encryption solutions, can address concerns over proprietary information, enabling broader collaboration across the scientific, business, and regulatory communities.

Ultimately, these data-sharing efforts and digital twin technologies can help increase the chances of success in clinical trials and biomanufacturing, creating a more efficient and cost-effective pathway for drug development and therapeutic innovations. We expect the biomanufacturing KH to be a data-driven learning platform to address different areas of biomedical engineering, being an essential tool for the translation of biomedical products that have complex processes (i.e., TERM products).

## 5. The Emergent Importance of Data Provenance for Management and Control of Chain of Identity (COI) in TERM Biomanufacturing

The recent development of TERM products, particularly for those that are designed for personalized therapies, presents numerous and significant operational challenges in the healthcare ecosystem. These challenges are driven by the dependency of these therapies on multiple modalities of advanced diagnostic and analytical data, genomic profiles, and the algorithmic design of the therapeutic.

Collectively, this emergent era of TERM therapeutics is characterized by 4 new criteria that the healthcare ecosystem has not evolved to accommodate including:

- High complexity operations: These End-to-End (E2E) operations are highly discontinuous and complex due to the extensive myriad of interdependent sub-processes, each with the potential to generate extensive and highly variable datasets.
- Multi-institutional: Multiple disparate institutions and entities are required to seamlessly integrate their subprocess across the E2E operation.
- Data-dependent efficacy: The efficacy of these therapies is dependent on error-free digital process control, traceability, and data provenance to ensure authenticated COI.
- Process variability: The processes across clinical, laboratory and bioproduction operations are highly variable due to the diversity of materials, cell systems, devices and formulations used in these processes
- Control & Ownership of COI Risk: The operational implementation and deployment of the E2E process requires capabilities for entity(ies) to have access to a myriad of data and controls that enable mitigation and ownership of the COI risk.

**Figure 5:**
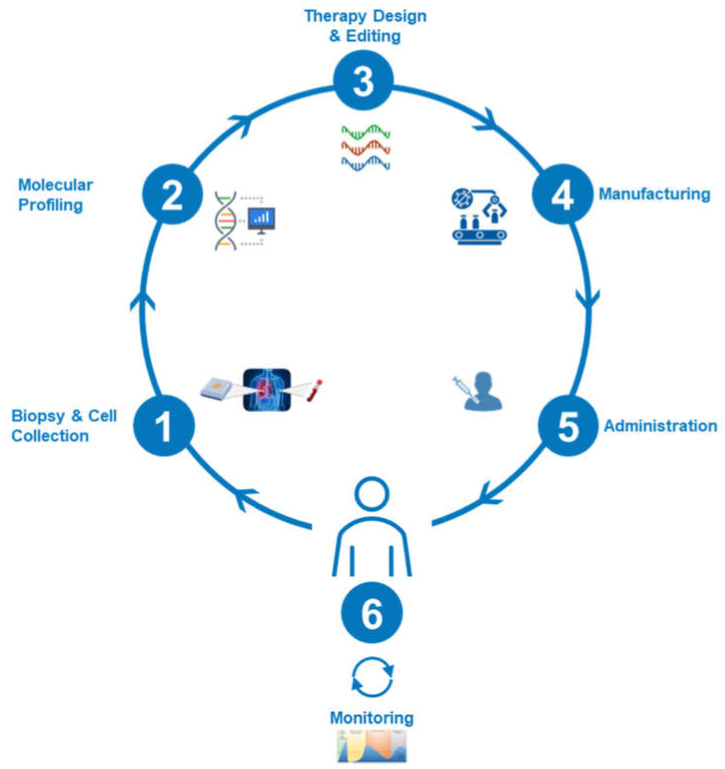
Generalized schema of the end-to-end operation in TERM biomanufacturing.

To illustrate the magnitude of divergence between current pharmaceutical E2E operations, we note that traditional pharmaceutical manufacturing operations are generally optimized to synthesize one therapeutic at scale that is delivered to millions of patients and generally achieves the status of blockbuster. Conversely, TERM therapies are often engineered at micro-scale that are effective only for a single patient, but must eventually be repeated across millions of patients on an error-free basis. A generalized schema of the E2E operation is illustrated below (Figure 5).

We assert that manufacturing of TERM therapies demands the need for the ultimate trust in the traceability and identities of biological materials that are obtained from patients and subsequently engineered for delivery to patients. This dependency ultimately falls to the mechanisms by which labware and clinical consumables are labeled across clinical, laboratory manufacturing operations, and supply chains. In fact, we foresee the need for a singular serialization technology that is interoperable across multiple disparate entities and also survives the harsh environmental exposures through the process. The need for digital process control for enabling COI is further validated in recent publications from regulatory bodies [64,65,66,67,68,69] that stipulate the need for:

1. Unique identifiers linking multiple products to a single therapy event
2. Handling and transport controls, barcoding, tracking and unique IDs
3. Mandates for labeling, tracking and identity documentation for human cellular and tissue-Based Products (HCT/Ps)
4. Validated systems for managing COI and chain of custody (COC)
5. Audit trails, data integrity and electronic controls

Current technologies such as barcodes and radio-frequency identification (RFID) are limited in a number of ways such as:

1. Visually readable barcodes on consumables can be corrupted, misread and cloned.
2. Adhesive labels on labware and clinical consumables typically do not survive harsh laboratory conditions and chemicals, such as organic solvents and extreme temperatures.
3. The small form factor of many consumables has limited surface availability to allow for multiple ID labels to be added by multiple institutions.

Collectively, these limitations present significant logistical challenges to organizations that require error-free implementation of personalized TERM therapies.

Industry participants, regulatory bodies and healthcare providers are proposing a singular multi-institutional serialization platform that solves these problems by embodying the following elements:

1. Tamper-proof identification that indelibly links patient samples and subsequent derivatives to their identity.
2. Immutable tracking to ensure that every operation subprocess is recorded in an unchangeable ledger
3. Interoperability across stakeholders that enable multi-institutional entities across manufacturers, hospitals, diagnostics laboratories, logistics providers, patient services centers and pharmaceutical companies to seamlessly verify the authenticity of the therapy and the E2E process.
4. Durability and size to enable tolerance for harsh environmental exposures and sufficient microscopic dimensions to be integrated with the small size of labware and clinical consumables.

Recent technologies such as cryptographic anchors, also known as crypto-anchors that interface with blockchain capabilities, are ideally suited to solve these challenges. They add tremendous value in securitizing the identity of biological specimens and materials, by being unclonable, incorruptible and unalterable when attached to a physical entity.

These allow the creation of a Digital Twin of the E2E operational process to ensure that the right patient receives the right therapy that was manufactured with right components under the right conditions by the right custodian. One example is applications in correcting medication errors that harm at least 1.5 million patients annually, with at least one death every day in the US. Incorrect medication type, doses, and administration directions are the most common dispensing medication errors that can be prevented by COI Technology (Milan, Italy) [70].

## 6. Knowledge Hub Case Studies

Here, we present two case studies to demonstrate how the KH can leverage the discussed data management technologies and support various applications in biomedical research and translation. The first case is a hypothetical scenario illustrating the creation and use of a KH for personalized stem cell therapy. The second case highlights a currently active pediatric database, Kidsights™ [25], which serves as an example of how the KH can be applied in practice to promote secure collaboration, foster innovation, and generate revenue.

### 6.1. Case 1: Personalized Stem Cell Therapy KH—A BT

Personalized stem cell therapy (PSCT), by its nature, is a complex and highly multidisciplinary approach. The data generated in this emerging field can immensely help our translational understanding of this technology and provides insights that collectively propel us forward quicker. Each step of the PSCT requires careful planning, execution, and adherence to technical, ethical and regulatory standards to ensure patient safety and treatment efficacy. The workflow of PSCT Knowledge Hub requires that each step is recorded on the blockchain and given a unique blockchain code, called Hashtag. Below are the steps involved in creating PSCT KH:

1. Patient Consultation and Initial Assessment
  - Patients consult with healthcare providers to assess their conditions and determine the need for stem cell therapy.
  - Patients opt into the Clinical Trial Advocacy Portal of the KH through their electronic health record profile (e.g., EPIC and CERNER).
  - Relevant medical history and conditions are recorded in a newly created block.
2. Stem Cell Source Identification
  - The source of stem cells (e.g., autologous, allogeneic) is determined. This is based on the patient’s condition, and specific therapeutic goals.
3. Informed Consent and Data Collection
  - Patients provide informed consent for data collection and treatment through EPIC or CERNER.
  - Patient specific data including genetic information, health records, and treatment preferences are added to the block.
4. Data Encryption and Hashing
  - All collected data is encrypted and hashed to ensure patient privacy and security.
  - This step maintains data integrity and confidentiality.
5. Personalized Treatment Plan Development
  - Based on the collected data and available therapeutic options, personalized treatment plans are developed for each patient and added to the block.
  - Treatment plans may include specific stem cell therapies tailored to the patient’s condition.
6. Treatment Preparation and Administration
  - Information on the used materials, technical protocols, and procedures for preparation of each patient’s therapeutic product is documented and assigned with COI tags and linked to the patient profile in the blockchain.
  - Stem cell therapy is administered to the patient based on the personalized treatment plan.
  - The administration procedure is documented on the blockchain, maintaining a complete history of treatments.
7. Post-Treatment Monitoring and Data Collection
  - Patients undergo monitoring to assess the effectiveness of the treatment.
  - Outcomes and side effects are recorded and encrypted on the blockchain for future reference.
8. Smart Contract Creation
  - Smart contracts are created for the block to govern the usage and sharing of patient data and the treatment outcomes.
  - Contracts specify conditions for data access and treatment protocols.
  - Smart contracts in the blockchain are executed automatically when the specified conditions are met, ensuring that all parties fulfill their obligations without manual intervention. If obligations are not met, depending on how the blockchain is administered, an “error” message is created, or a no execution command will be initiated meaning the process will not proceed to the next step or the whole process can revert to the beginning.
9. Stem Cell Processing and Quality Control
  - Stem cells are processed in a controlled environment, and in some cases by multiple commercial and academic stakeholders. COI data from the blockchain provides documentation of this complex process for every personalized therapy.
  - Quality control measures are implemented, and results are recorded on the blockchain for transparency.
  - Biomanufacturing environmental data can also be included on the blockchain to ensure proper handling guidelines are met. As with smart contracts, deviations in environmental conditions or product preparation conditions, recorded on the blockchain, can send alerts and a series of appropriate protocols can be implemented.
  - Lab errors that could cause unique patient biological materials to be mixed up are also eliminated.
10. Data Storage on Blockchain
  - Encrypted patient data, treatment protocols, and quality control results are stored on the blockchain.
  - This creates an immutable record that can be accessed by authorized parties.
11. Research and Development
  - Authorized researchers can access and analyze this immutable record to study the anonymized data with the goal of improving stem cell therapies.
  - Insights from research are fed back into the treatment protocols.
12. Feedback Loop for Continuous Improvement
  - Continuous collection of data from treated patients helps refine stem cell therapies and treatment protocols.
  - This feedback loop allows for ongoing research and development based on real-world outcomes.
13. Patient Control and Data Access
  - Patients can control access to their data and choose to share their treatment outcomes with researchers or providers.
  - The use of smart contracts ensures that data sharing is secure and compliant with regulations.
  - Patient outcomes could also be linked with manufacturing processes.
14. Regulatory Compliance
  - Throughout the workflow, compliance with healthcare regulations (like HIPAA, GDPR) is maintained.
  - Blockchain provides a transparent and traceable record of data handling.

### 6.2. Case 2: Kidsights^TM^ Pediatric Database—A FL and TripleBlind Technology

Kidsights^TM^ is a wholly owned subsidiary of Gillette Children’s hospital (Saint Paul, MN, USA) and its purpose is to create a consortium of hospitals that pool their data and make the data available to businesses, researchers and individuals in a completely privacy-preserving way. KidSights™ launched in 2023 by Gillette Children’s—an established U.S.-based specialty pediatric hospital—and it has quickly expanded to include internationally renowned partners. Holland Bloorview Kids Rehabilitation Hospital in Toronto, Canada, recently became the first Canadian member, making two founding members of the consortium so far. Although formal publications or clinical validations stemming from KidSights are not yet publicly detailed, the program’s design empowers pediatric innovation by offering researchers and commercial developers access to aggregated, deidentified Electronic Medical Record (EMR)-based real-world data. This structure lays a solid foundation for future studies and commercialization efforts tailored to the needs of children with complex and rare medical conditions. KidSights participates in rigorous data governance and privacy compliance, adhering to Canadian and U.S. privacy laws including PHIPA, PIPEDA, and HIPAA. It employs advanced de-identification techniques to support secure, privacy-preserving usage of clinical data for research, therapeutic innovation, and systems-level insight. Table 2 presents a KidSights^TM^ overview.

Kidsights^TM^ employs the TripleBlind technology discussed earlier to allow data users to leverage the data without taking possession of the data, while also employing effective technical limiters on the content that a data user can receive (i.e., what knowledge they can take out of the federated pool). Several children’s specialty hospitals contribute to the data pool, at least one of which is outside the US.

The consortium wants to “supply everyday insights into pediatric specialty and rare diseases” [25]. Privacy is especially difficult to maintain when dealing with rare disease in children. The cases can be so rare as to only occur once in a city, thus making the data almost impossible to truly de-identify, especially if the data is combined with normal “social determinants of medicine” or locational data. The privacy enhancing technologies employed by Kidsights^TM^ accommodate both extensive research and privacy.

Kidsights^TM^ data users run the gamut of researchers to technical innovators. Once privacy is ensured, the uses for the data are limitless. In some cases, data consumers are commercial innovators looking to accelerate the development of specialized equipment (i.e., entrepreneurs), and they may be seeking something as simple as a general market sizing. In other cases, the data users are researchers looking for data to train AI diagnostic agents to assist doctors with diagnoses. Additionally, the data users might be the consortium members themselves benchmarking each other’s performance. And there are many instances of pharmaceutical companies using the data to prepare and execute clinical trials. In each case the consortium has a revenue model that appropriately awards the hospitals that allow their data to be used.

There are several essential points in this case study. First, the hospitals do not move their data to a “central database”, the data stays behind the various hospital’s firewalls. Second, the technology supports data privacy across international jurisdiction. Third, each hospital can independently decide whether or not to allow their data to be used for any request. Fourth, the tool set allows the data to be used for almost any aggregated query, machine learning, statistical, analytical or artificial intelligence building/test purpose. The uses of the data can be as creative as the users require.

Overall, Kidsights^TM^ is speeding up the pace of innovation in children’s health and driving additional revenues to the various member institutions.

## 7. Vision for Knowledge Hub and Next Steps

**Figure 6:**
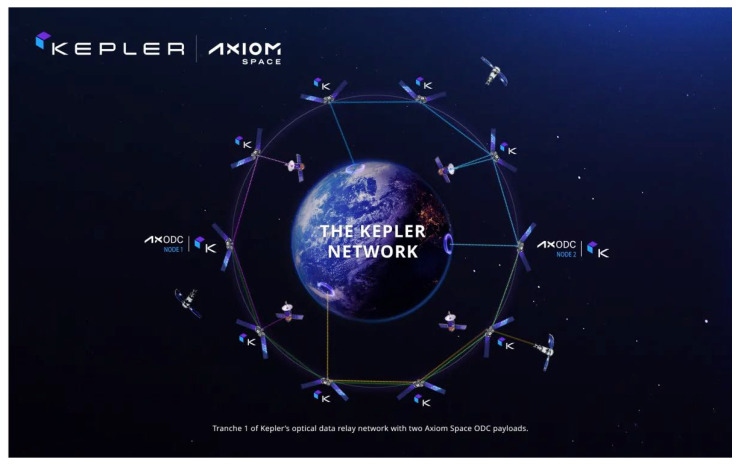
Schematic of how data could be connected at a global scale to include information from LEO. In Space Manufacturing for terrestrial benefit is going to be a disruptive technology platform that will propel both life and material science into the future. It will have huge societal benefits. KH will accelerate this platform with compute on the edge that is enabled by networks such at Axiom Kepler’s Orbital Data Center [71]. Courtesy of Axiom Space and Kepler Communications. The figure illustrates the Kepler Network, an orbital mesh of interconnected satellites equipped with optical inter-satellite links (depicted as lines connecting nodes) that enable high-speed, low-latency data relay across space. The highlighted Axiom ODC (Orbital Data Center) nodes represent [71] facilitate scalable, automated operations in low Earth orbit and beyond.

Our vision KH is to establish a data-driven learning platform for biomanufactured products—connecting patients, scientists, clinicians, international regulatory authorities, and companies to accelerate development and minimize redundancies as well as providing a pathway for global regulatory harmonization. We aim to create a global, dynamic biomanufacturing registry powered by smart contracts, quality and safety certificates, and decentralized data architectures. This registry would support a highly modular manufacturing workflow capable of pooling from any certified cell bank, gene engineering platform, or GMP-grade biomaterial—whether produced terrestrially or in low Earth orbit (LEO). As illustrated in Figure 6, orbital data infrastructure, such as the Axiom Space–Kepler Communications network, provides the technical foundation to link space-based manufacturing nodes with terrestrial stakeholders [71].

To make this vision operational, the KH must integrate orbital data centers with terrestrial cloud platforms in a secure, scalable, and latency-aware architecture. These orbital compute nodes will not only support in situ analysis and autonomous process control but also facilitate encrypted exchange with Earth-based regulatory, commercial, and scientific systems. Web 3.0 technologies, including blockchain and smart contracts, will govern access, automate compliance, and coordinate execution across multi-party workflows [72,73]. Smart contracts can, for example, release data or activate manufacturing processes in response to verified triggers, with embedded AI/ML models enabling real-time decision-making and adaptive control [72].

Terrestrial Manufacturing for biologics and advanced materials is hitting the wall because of gravity. This is due to the following phenomena: container interactions, thermal convection, flotation & sedimentation and hydrostatic pressure, which do not exist or are significantly reduced in low Earth Orbit (LEO) [74]. Because of this in the next 3–10 years Biomanufacturing in Space is going to be a disruptive technology platform. The environment will provide new drug delivery mechanisms due to pure crystal formation, artificial retinas, as well as the production of pluripotent stem cells and bio inks, which are examples of how microgravity can stimulate the medical and bioscience industries [75].

IP can be managed through patent pools, while manufacturing rights and data assets can be tokenized, opening new models for licensing, collaboration, and revenue sharing [76]. Furthermore, cryptographic technologies such as FHE and Trusted Execution Environments (TEEs) will ensure privacy-preserving computation and secure governance of sensitive genomic, cellular, or proprietary process data [77,78]. These technologies allow stakeholders to collaborate across jurisdictional, commercial, and geopolitical boundaries without compromising security or control.

In addition, we will develop a KH monetization model. New revenue models for biomanufacturing KH, incentivizing data and model sharing via a biomanufacturing KH represents a pivotal mechanism for accelerating innovation and commercialization in biomanufacturing. By treating datasets and AI/ML models as knowledge assets, it becomes feasible to construct revenue models analogous to those in digital content economies—where creators are compensated for the downstream use of their contributions [76]. In this paradigm, data generators (e.g., research institutions, clinical laboratories, manufacturing facilities) and model developers (e.g., algorithm designers, computational biologists) serve as producers of high-value digital assets. These assets are then accessed, licensed, or consumed by downstream users, including biotech firms, regulatory consultants, and process engineers—who derive measurable value from their application. Revenue is generated via usage-based pricing, subscription models, or performance-based royalties [73], ensuring that asset creators receive financial recognition and sustainable incentives. This economic structure has the potential to overcome entrenched data silos by aligning individual or institutional benefit with collective advancement. More importantly, it offers a scalable and equitable framework to support continuous innovation and expedite the translation of insights into deployable biomanufacturing solutions. In the context of emerging public–private consortia and precompetitive data-sharing initiatives, such as the National Institute for Innovation in Manufacturing Biopharmaceuticals (NIIMBL), these models are not only feasible but increasingly necessary to achieve national strategic goals in biotechnology [72].

Our approach for KH monetization is inspired by two successful existing models from other industries: (1) the Internet Engineering Task Force (IETF); and (2) the Via Licensing Alliance. The IETF coordinates the formation of voluntary technical standards to maintain and improve the usability and interoperability of the Internet. These consensus-driven standards define the underlying foundation that enables an array of e-commerce and digital services companies to share data and engage in transactions. The Via Licensing Alliance is the world’s largest independent administrator of patent pools and provides licensors and licensees a common platform to deliver one-stop shopping for essential IP.

We envision the future of regenerative medicine supporting open collaboration and pro-competitive innovation in a similar way to the IETF and the Via Licensing Alliance through a diversified set of revenue streams. Our concept features a tiered membership structure with fees for industry, academic, and nonprofit participants, alongside sponsorship opportunities for events and working groups. Additional revenue streams include standards certification programs, field-of-use licensing via shared IP pools, and value-added subscription services such as analytics, regulatory guidance, and VR-based training. A privacy-preserving data exchange system enables monetization while protecting contributors, and additional support comes from grants, government contracts, and open-access publishing. This multi-lever approach ensures long-term sustainability while incentivizing engagement and innovation.

Ultimately, the KH represents a convergence of biotechnology, space systems engineering, cryptography, and decentralized computing. It will enable the collaborative development of novel biomanufactured products—including those uniquely enabled by microgravity conditions—at a scale and speed previously unimaginable. By connecting orbital biomanufacturing capability with Earth-based knowledge systems, the KH can serve as the nexus for a new class of globally coordinated, secure, and innovation-driven life science supply chains.

## Figures and Tables

**Table 1:** Existing regulations to ensure data protections and privacy.

Region	Regulations	Focus Areas
European Union	GDPR (General Data Protection Regulation) [6]	Classifies healthcare data as sensitive data with additional protection rules
	NIS2 Directive (effective since 2024) [7]	Aims to reduce cybersecurity risks for healthcare institutions
United States	HIPAA (Health Insurance Portability and Accountability Act of 1996) [8]	Regulates use and disclosure of patients’ health information
	HITECH Act (2009) [8]	Promotes the adoption and meaningful use of health information technology
	HIPAA Final Omnibus Rule (2013) [8]	Strengthens privacy and security protections for health information
Canada	PIPEDA [9]	Complex, multi-jurisdictional framework with 29 + privacy statutes Governs federal and interprovincial data privacy

**Table 2:** KidSights^TM^ overview.

Feature	Current Status
Consortium Membership	Gillette Children’s (U.S.) and Holland Bloorview (Canada)
Scale of Use	Two leading pediatric specialty institutions
Outputs & Publications	No publicly disclosed publications or validated outputs yet
Data Access & Use	Aggregated de-identified EMR data available to researchers and innovators
Privacy & Security	Compliance with PHIPA, PIPEDA, HIPAA; advanced de-identification

## Abbreviations

The following abbreviations are used in this manuscript:

AI	Artificial Intelligence
API	Application Programming Interface
CIRM	California Institute of Regenerative Medicine
COC	Chain of Custody
COI	Chain of Identity
CT	Computer Tomography
DeFi	Decentralized Finance
DHDM	Digital Health Data Marketplace
DLT	Distributed Ledger Technology
DRM	Digital Rights Management
ECG	Electrocardiogram
EDA	Exploratory Data Analysis
EMR	Electronic Medical Record
EU	European Union
E2E	End-to-End
FRAND	Fair, Reasonable, and Non-Discriminatory
FDA	Food and Drug Administration
FL	Federated Learning
FHE	Fully Homomorphic Encryption
GDPR	General Data Protection Regulation
GMP	Good Manufacturing Practices
GUI	Graphical User Interface
HCT/Ps	Human Cellular Tissue-based Products
HIPAA	Health Insurance Portability and Accountability Act
IBC	Inter-Blockchain Communication
IETF	Internet Engineering Task Force
IIoT	Industrial-internet-of-things
IP	Intellectual Property
KH	Knowledge Hub
LEO	Low Earth Orbit
MPC	Multi-party computation
ML	Machine Learning
NAM	New Approach Methodologies
NIH	National Institutes of Health
NIIMB	National Institute for Innovation in Manufacturing Biopharmaceuticals
ODC	Orbital Data Center
PSCT	Personalized Stem Cell Therapy
RFID	Radio Frequency Identification
RMMS	Regenerative Medicine Manufacturing Society
R&D	Research and Development
TERM	Tissue Engineering and Regenerative Medicine
TEE	Trusted Execution Environments
UNCTAD	United Nations Trade and Development
